# Supplementary material for: Receptor-mediated cargo hitchhiking on bulk autophagy
Source: EMBO J. 2024 May 16;43(15):3. doi: 10.1038/s44318-024-00091-8 (PMC11294605; doi:10.1038/s44318-024-00091-8)
Supplement: Supplementary file 1 — Appendix [file 44318_2024_91_MOESM1_ESM.pdf]

# Appendix for
# Receptor-mediated cargo hitchhiking on bulk autophagy

Table of Contents | Page


| Table of Contents | Page |
|---|---|
| Appendix Fig S1 | 1 |
| Appendix Fig S2 | 2 |
| Appendix Fig S3 | 3 |

# Appendix Figure S1

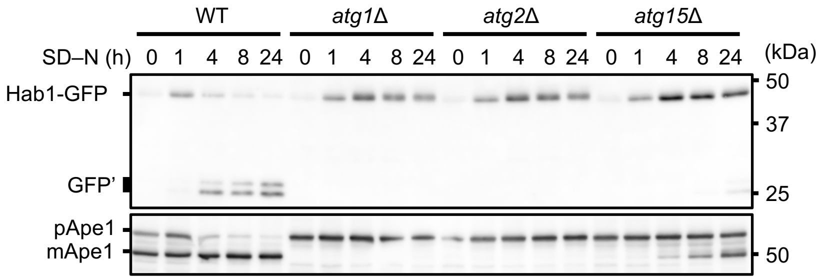


**Appendix Figure S1 Hab1-GFP cleavage during nitrogen-starved cells (related Fig 4)**

GFP cleavage assay of Hab1-GFP-expressing WT, *atg1*Δ, *atg2*Δ, and *atg15*Δ cells at indicated time points of nitrogen starvation. Related to Fig 2C.

# Appendix Figure S2

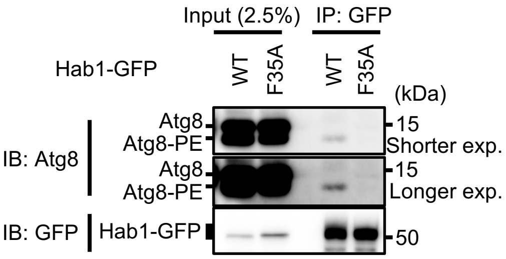


**Appendix Figure S2 Full-length Hab1 also binds specifically to Atg8-PE (related Fig 2C)**

Binding of Atg8 to Hab1-GFP assessed by immunoprecipitation. Samples were obtained from cells treated with rapamycin and PMSF (1 mM) for 1 h before being subjected to immunoblotting.

# Appendix Figure S3

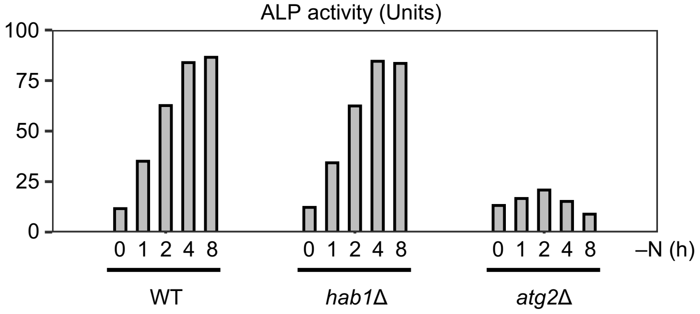


**Appendix Figure S3 Hab1 does not involved in bulk autophagy activity**

Measurement of autophagy activity by ALP assay in Pho8Δ60 expressing WT, *hab1*Δ, and *atg2*Δ cells.
