# Supplementary figures and images for: Receptor-mediated cargo hitchhiking on bulk autophagy
Source: EMBO J. 2024 May 16;43(15):3. doi: 10.1038/s44318-024-00091-8 (PMC11294605; doi:10.1038/s44318-024-00091-8)

Figure 1B

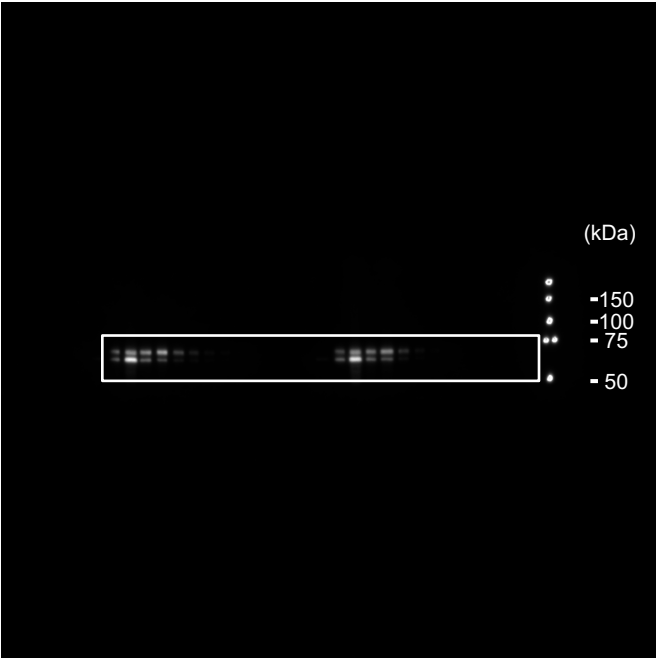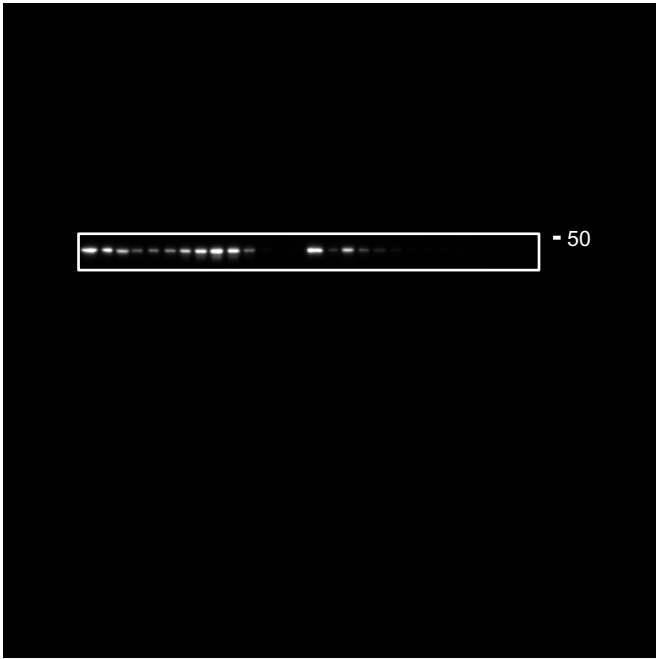

Supplement: Supplementary file 7 — Source data Fig. 1 [file 44318_2024_91_MOESM7_ESM.zip › EMBOJ-2023-115374R1_SourceDataForFig1/1B/1B.pdf]

Figure 2A

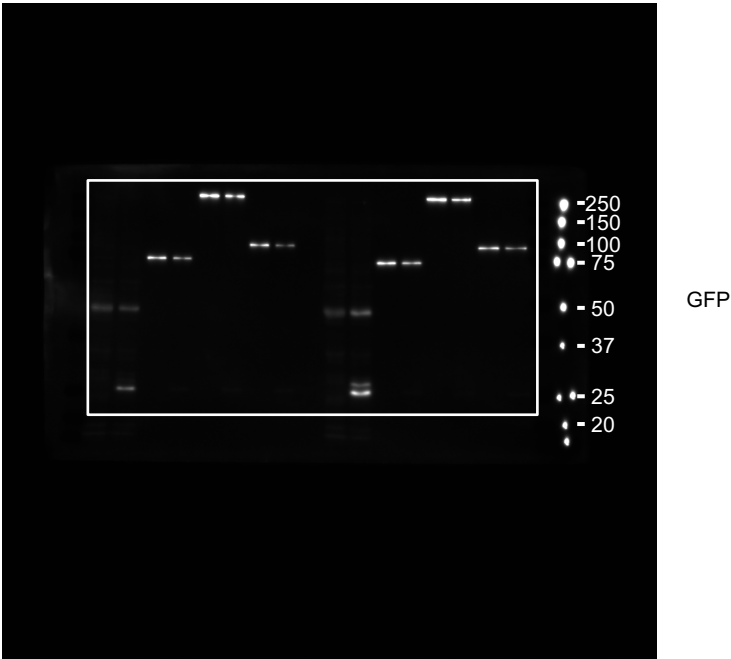

Supplement: Supplementary file 8 — Source data Fig. 2 [file 44318_2024_91_MOESM8_ESM.zip › EMBOJ-2023-115374R1_SourceDataForFig2/2A/2A.pdf]

Figure 2C

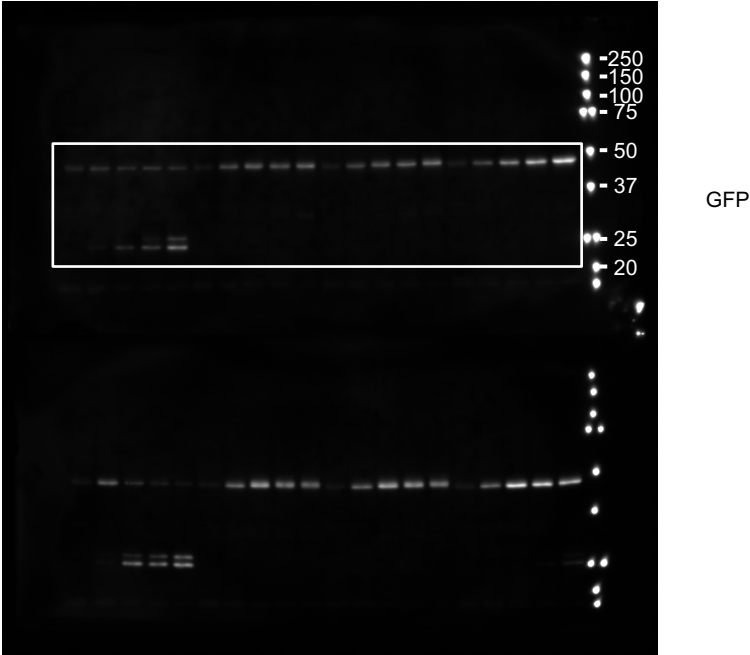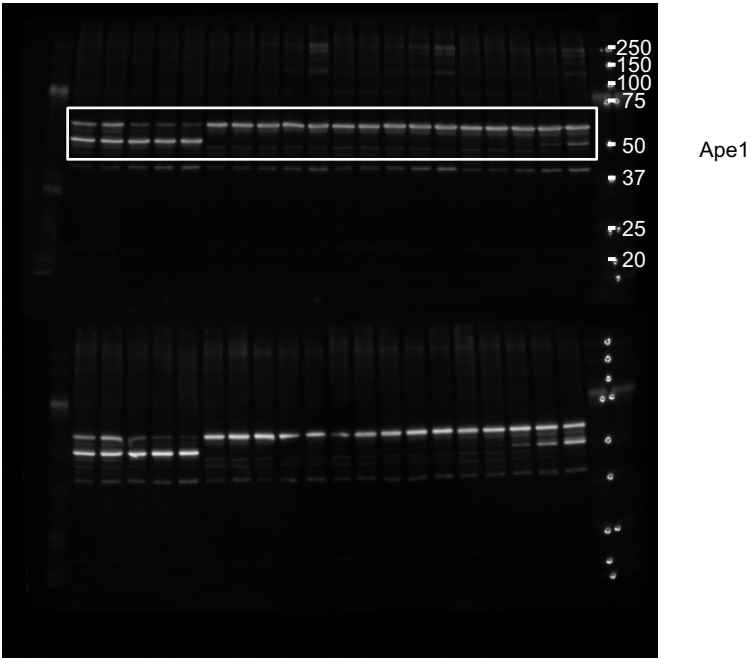

Supplement: Supplementary file 8 — Source data Fig. 2 [file 44318_2024_91_MOESM8_ESM.zip › EMBOJ-2023-115374R1_SourceDataForFig2/2C/2C.pdf]

Figure 2D

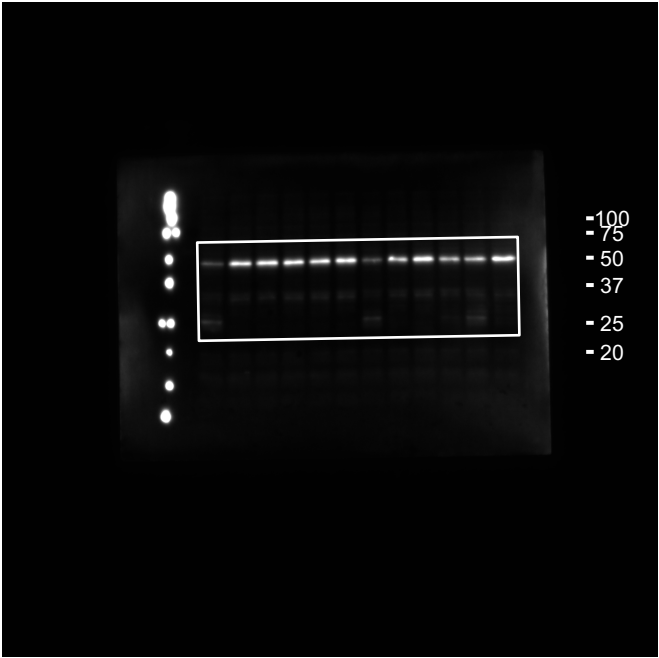

GFP

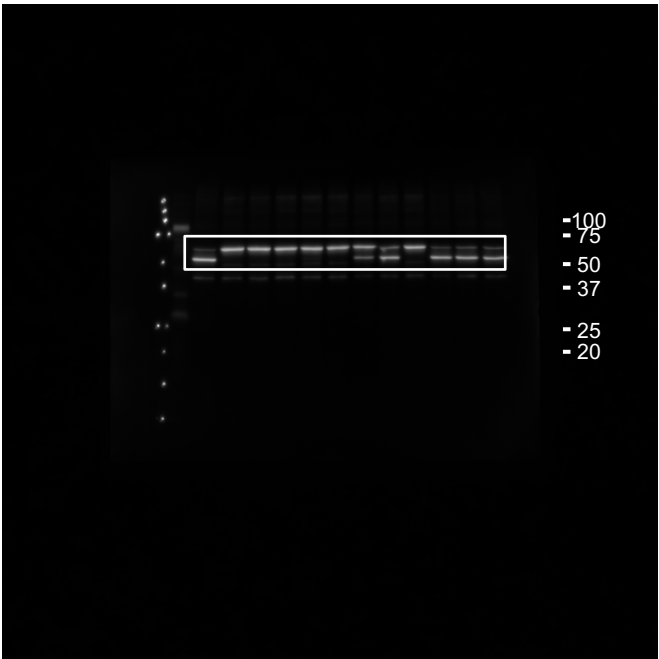

Ape1

Supplement: Supplementary file 8 — Source data Fig. 2 [file 44318_2024_91_MOESM8_ESM.zip › EMBOJ-2023-115374R1_SourceDataForFig2/2D/2D.pdf]

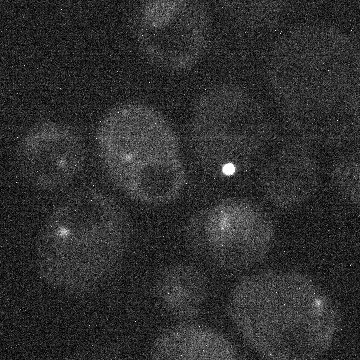

Supplement: Supplementary file 8 — Source data Fig. 2 [file 44318_2024_91_MOESM8_ESM.zip › EMBOJ-2023-115374R1_SourceDataForFig2/2B/2, WT 1h GFP.tif]

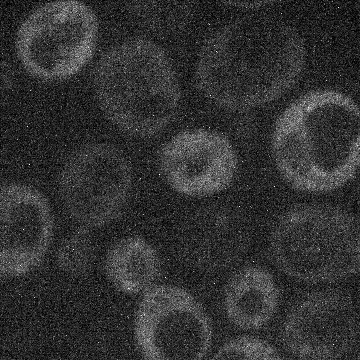

Supplement: Supplementary file 8 — Source data Fig. 2 [file 44318_2024_91_MOESM8_ESM.zip › EMBOJ-2023-115374R1_SourceDataForFig2/2B/5, atg2 1h GFP.tif]

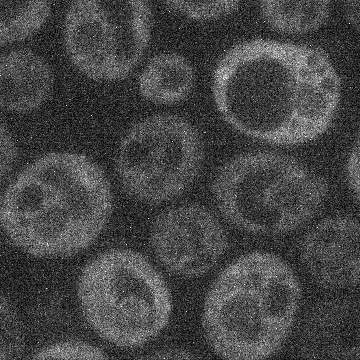

Supplement: Supplementary file 8 — Source data Fig. 2 [file 44318_2024_91_MOESM8_ESM.zip › EMBOJ-2023-115374R1_SourceDataForFig2/2B/6, atg2 4h GFP.tif]

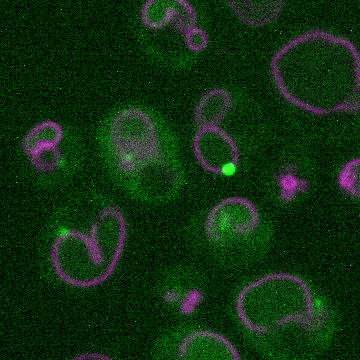

Supplement: Supplementary file 8 — Source data Fig. 2 [file 44318_2024_91_MOESM8_ESM.zip › EMBOJ-2023-115374R1_SourceDataForFig2/2B/2, WT 1h merge.tif]

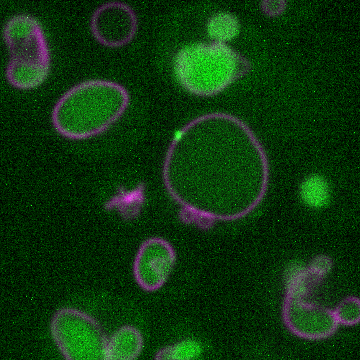

Supplement: Supplementary file 8 — Source data Fig. 2 [file 44318_2024_91_MOESM8_ESM.zip › EMBOJ-2023-115374R1_SourceDataForFig2/2B/3, WT 4h merge.tif]

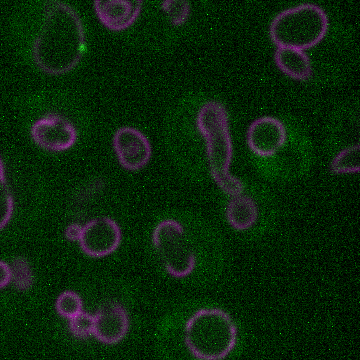

Supplement: Supplementary file 8 — Source data Fig. 2 [file 44318_2024_91_MOESM8_ESM.zip › EMBOJ-2023-115374R1_SourceDataForFig2/2B/1, WT 0h merge.tif]

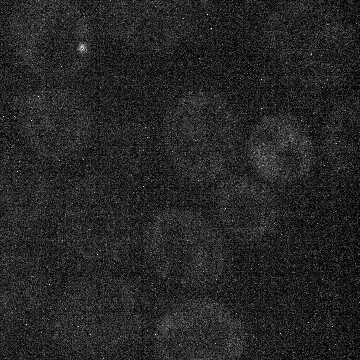

Supplement: Supplementary file 8 — Source data Fig. 2 [file 44318_2024_91_MOESM8_ESM.zip › EMBOJ-2023-115374R1_SourceDataForFig2/2B/1, WT 0h GFP.tif]

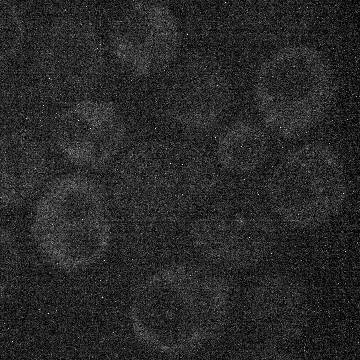

Supplement: Supplementary file 8 — Source data Fig. 2 [file 44318_2024_91_MOESM8_ESM.zip › EMBOJ-2023-115374R1_SourceDataForFig2/2B/4, atg2 0h GFP.tif]

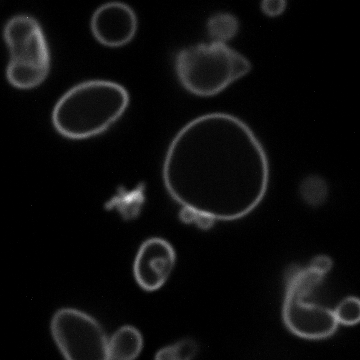

Supplement: Supplementary file 8 — Source data Fig. 2 [file 44318_2024_91_MOESM8_ESM.zip › EMBOJ-2023-115374R1_SourceDataForFig2/2B/3, WT 4h mCh.tif]

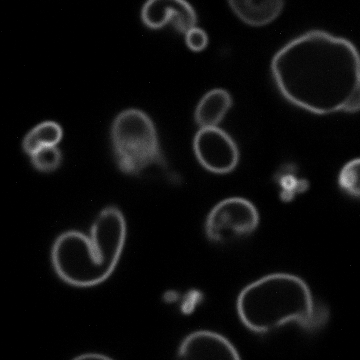

Supplement: Supplementary file 8 — Source data Fig. 2 [file 44318_2024_91_MOESM8_ESM.zip › EMBOJ-2023-115374R1_SourceDataForFig2/2B/2, WT 1h mCh.tif]

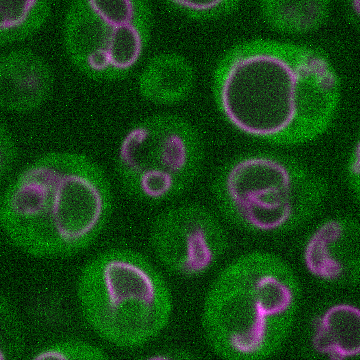

Supplement: Supplementary file 8 — Source data Fig. 2 [file 44318_2024_91_MOESM8_ESM.zip › EMBOJ-2023-115374R1_SourceDataForFig2/2B/6, atg2 4h merge.tif]

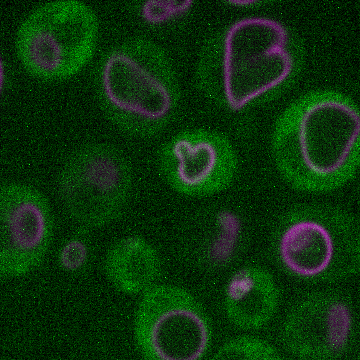

Supplement: Supplementary file 8 — Source data Fig. 2 [file 44318_2024_91_MOESM8_ESM.zip › EMBOJ-2023-115374R1_SourceDataForFig2/2B/5, atg2 1h merge.tif]

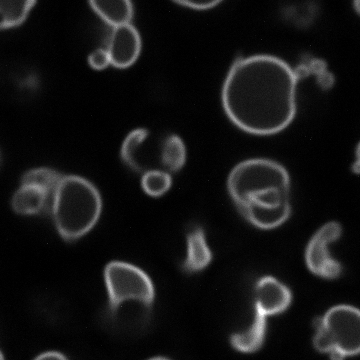

Supplement: Supplementary file 8 — Source data Fig. 2 [file 44318_2024_91_MOESM8_ESM.zip › EMBOJ-2023-115374R1_SourceDataForFig2/2B/6, atg2 4h mCh.tif]

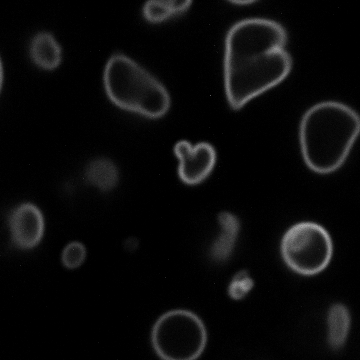

Supplement: Supplementary file 8 — Source data Fig. 2 [file 44318_2024_91_MOESM8_ESM.zip › EMBOJ-2023-115374R1_SourceDataForFig2/2B/5, atg2 1h mCh.tif]

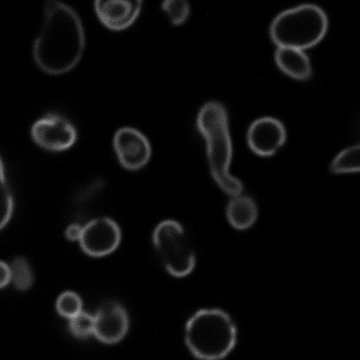

Supplement: Supplementary file 8 — Source data Fig. 2 [file 44318_2024_91_MOESM8_ESM.zip › EMBOJ-2023-115374R1_SourceDataForFig2/2B/1, WT 0h mCh.tif]

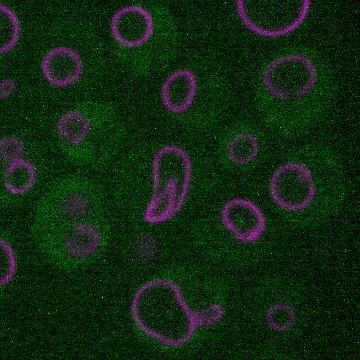

Supplement: Supplementary file 8 — Source data Fig. 2 [file 44318_2024_91_MOESM8_ESM.zip › EMBOJ-2023-115374R1_SourceDataForFig2/2B/4, atg2 0h merge.tif]

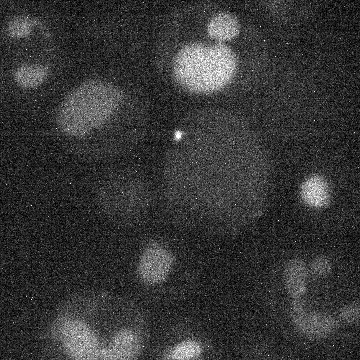

Supplement: Supplementary file 8 — Source data Fig. 2 [file 44318_2024_91_MOESM8_ESM.zip › EMBOJ-2023-115374R1_SourceDataForFig2/2B/3, WT 4h GFP.tif]

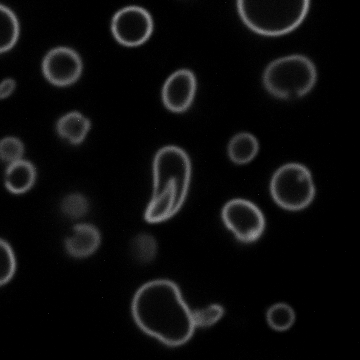

Supplement: Supplementary file 8 — Source data Fig. 2 [file 44318_2024_91_MOESM8_ESM.zip › EMBOJ-2023-115374R1_SourceDataForFig2/2B/4, atg2 0h mCh.tif]

Figure 3E

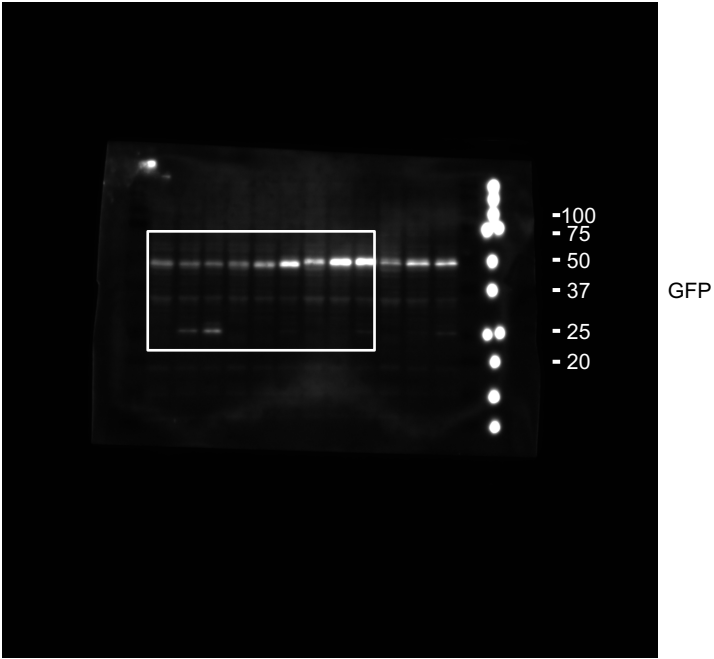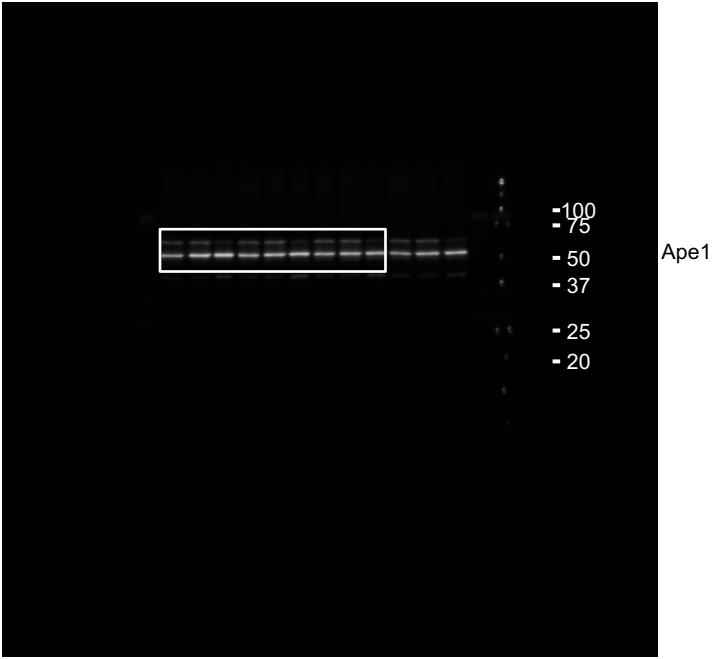

Supplement: Supplementary file 9 — Source data Fig. 3 [file 44318_2024_91_MOESM9_ESM.zip › EMBOJ-2023-115374R1_SourceDataForFig3/3E/3E.pdf]

Figure 3B

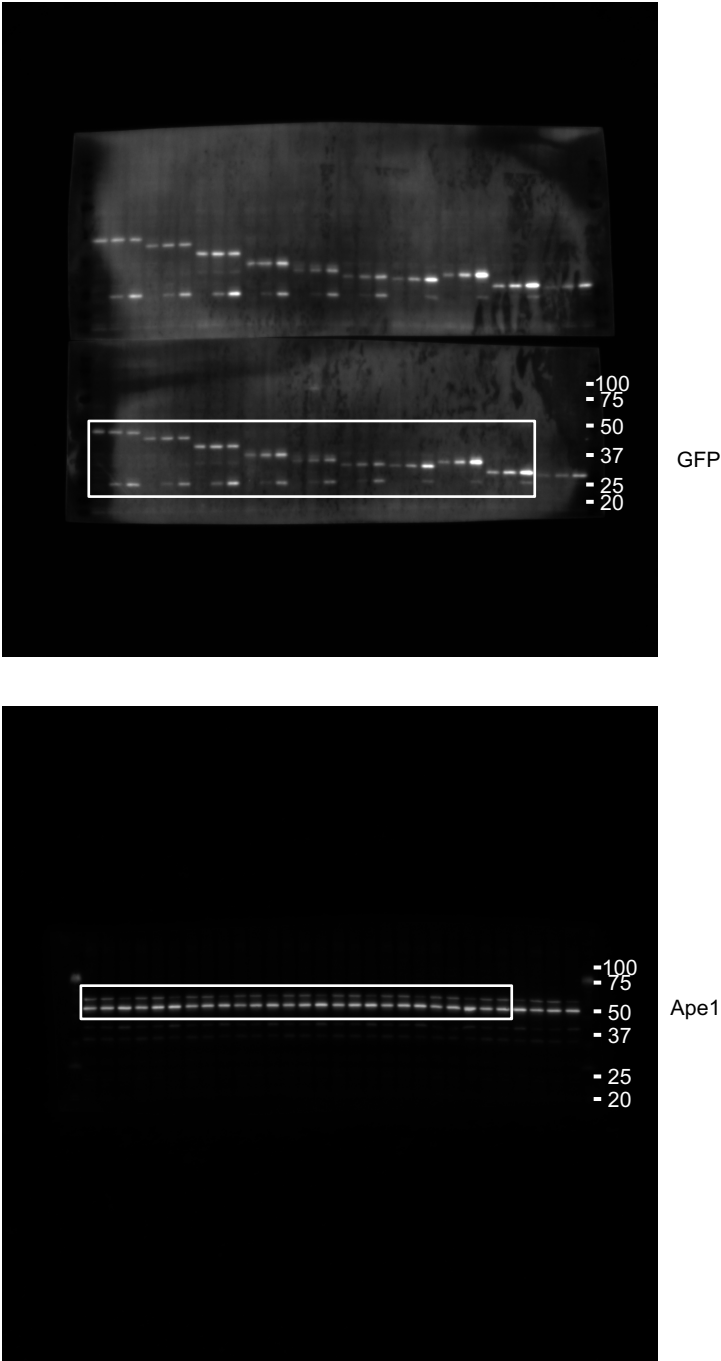

Supplement: Supplementary file 9 — Source data Fig. 3 [file 44318_2024_91_MOESM9_ESM.zip › EMBOJ-2023-115374R1_SourceDataForFig3/3B/3B.pdf]

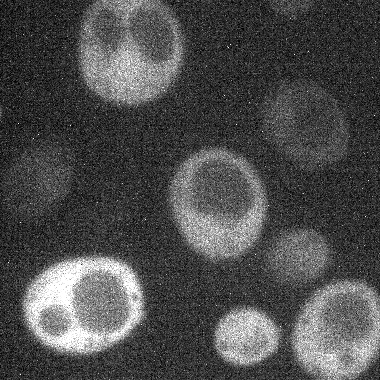

Supplement: Supplementary file 9 — Source data Fig. 3 [file 44318_2024_91_MOESM9_ESM.zip › EMBOJ-2023-115374R1_SourceDataForFig3/3D/4, Hab1(1-18, 3A)-GFP.tif]

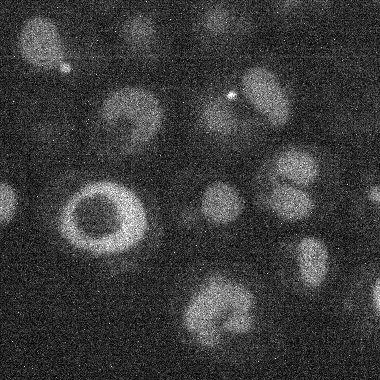

Supplement: Supplementary file 9 — Source data Fig. 3 [file 44318_2024_91_MOESM9_ESM.zip › EMBOJ-2023-115374R1_SourceDataForFig3/3D/1, Hab1(1-144)-GFP.tif]

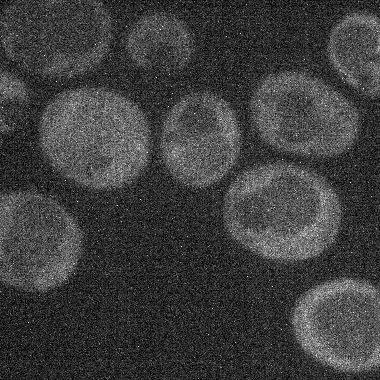

Supplement: Supplementary file 9 — Source data Fig. 3 [file 44318_2024_91_MOESM9_ESM.zip › EMBOJ-2023-115374R1_SourceDataForFig3/3D/2, Hab1(1-144, 3A)-GFP.tif]

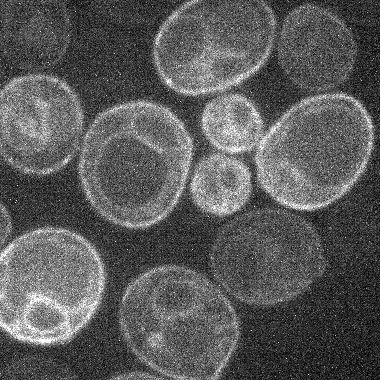

Supplement: Supplementary file 9 — Source data Fig. 3 [file 44318_2024_91_MOESM9_ESM.zip › EMBOJ-2023-115374R1_SourceDataForFig3/3D/3, Hab1(1-18)-GFP.tif]

Figure 3G

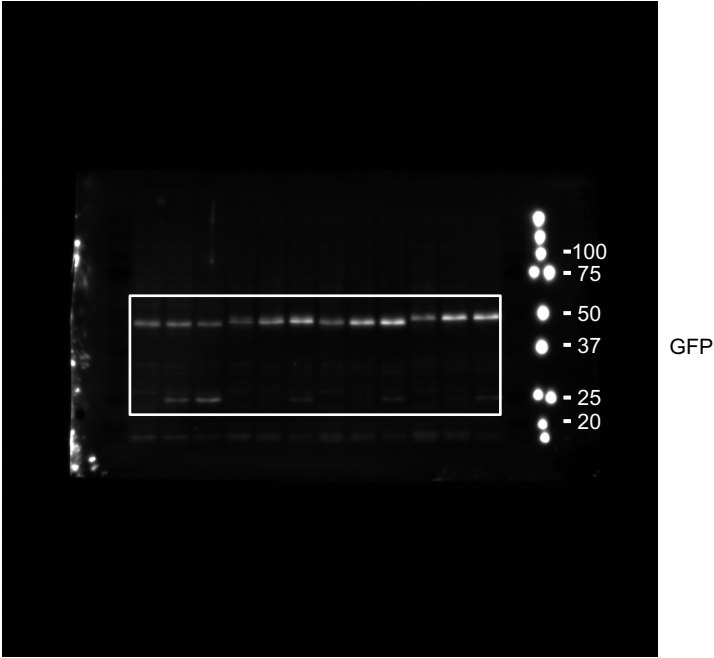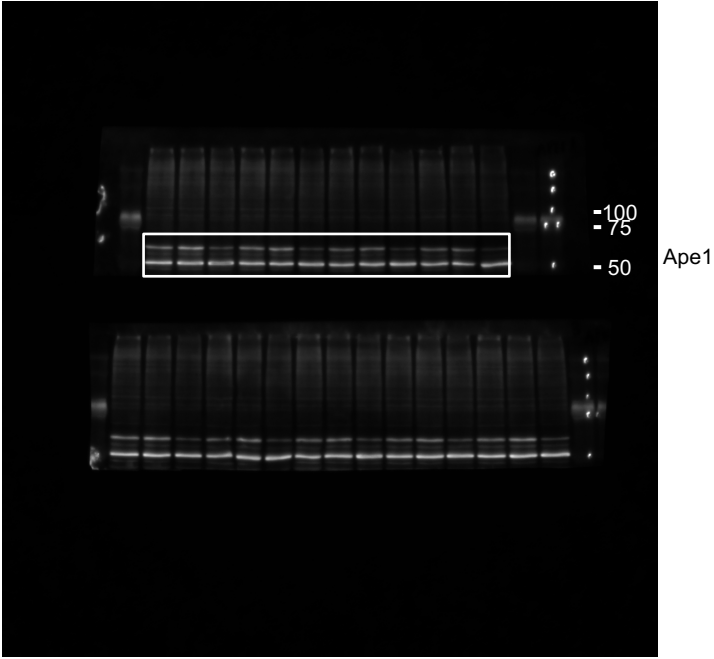

Supplement: Supplementary file 9 — Source data Fig. 3 [file 44318_2024_91_MOESM9_ESM.zip › EMBOJ-2023-115374R1_SourceDataForFig3/3G/3G.pdf]

Figure 4B

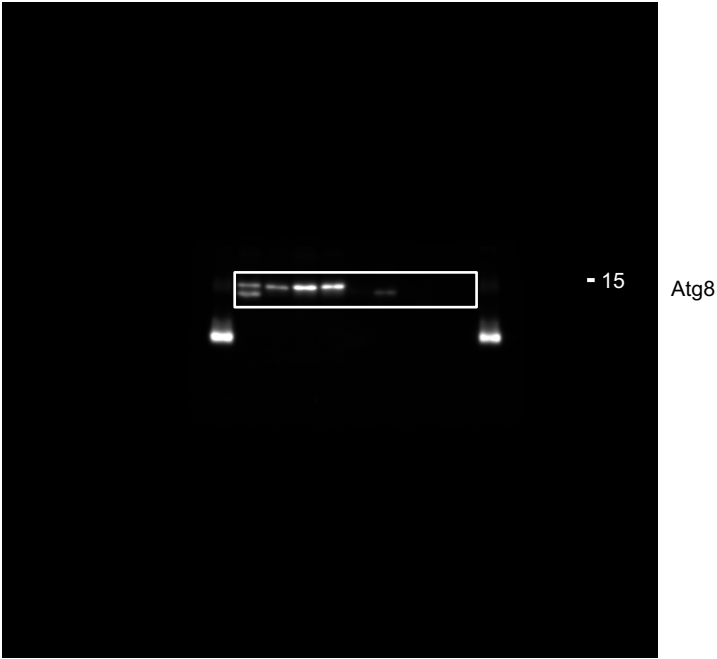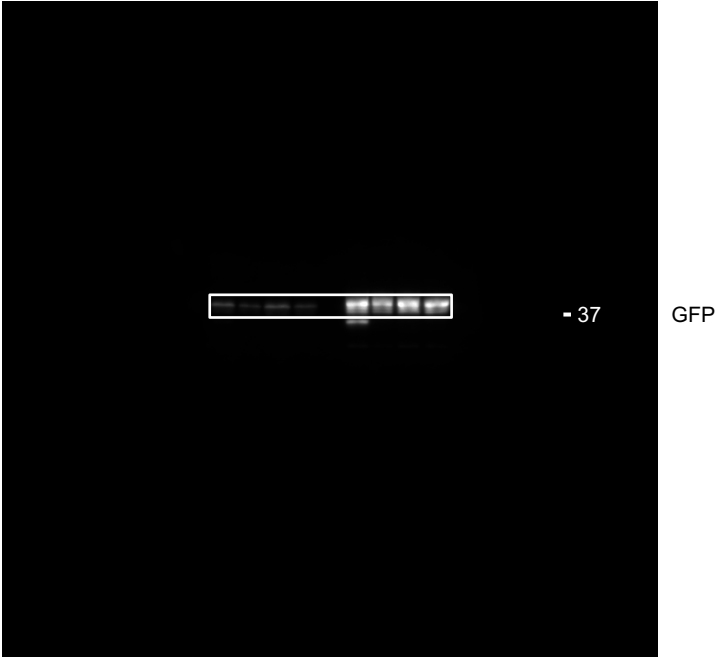

Supplement: Supplementary file 10 — Source data Fig. 4 [file 44318_2024_91_MOESM10_ESM.zip › EMBOJ-2023-115374R1_SourceDataForFig4/4B/4B.pdf]

Figure 4C

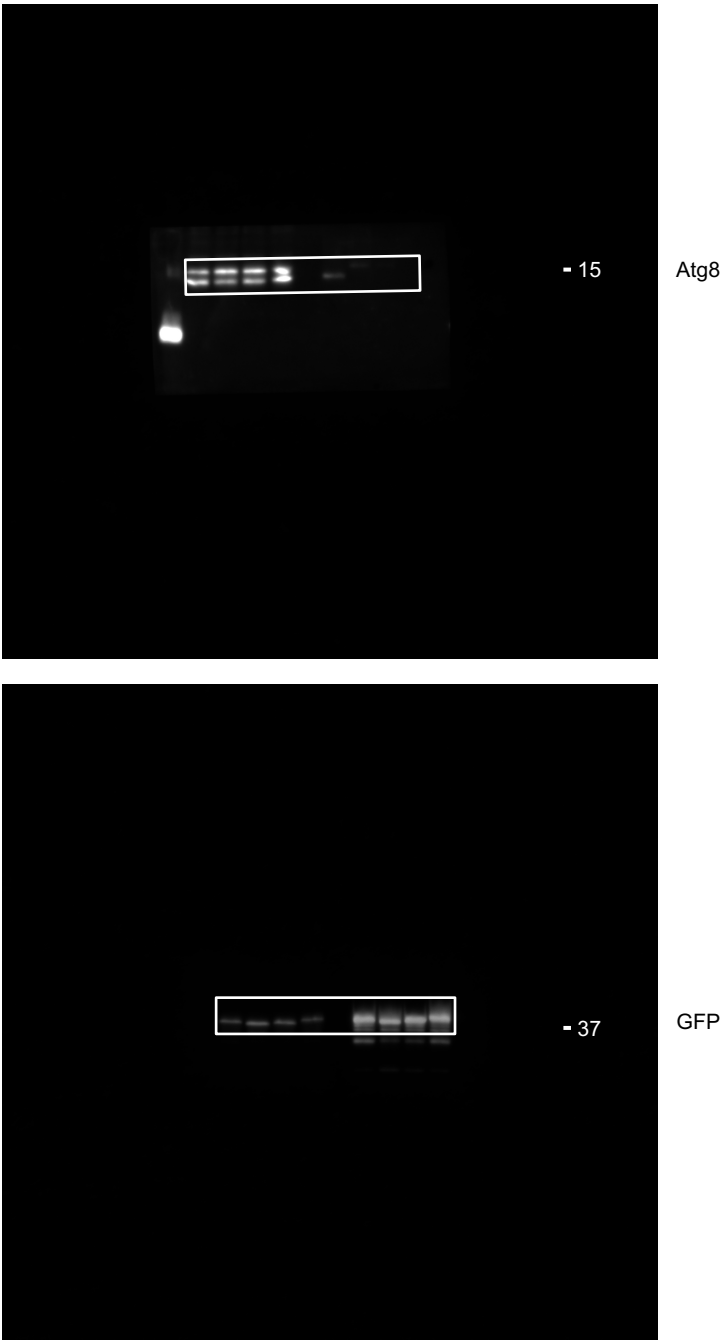

Supplement: Supplementary file 10 — Source data Fig. 4 [file 44318_2024_91_MOESM10_ESM.zip › EMBOJ-2023-115374R1_SourceDataForFig4/4C/4C.pdf]

Figure 4A

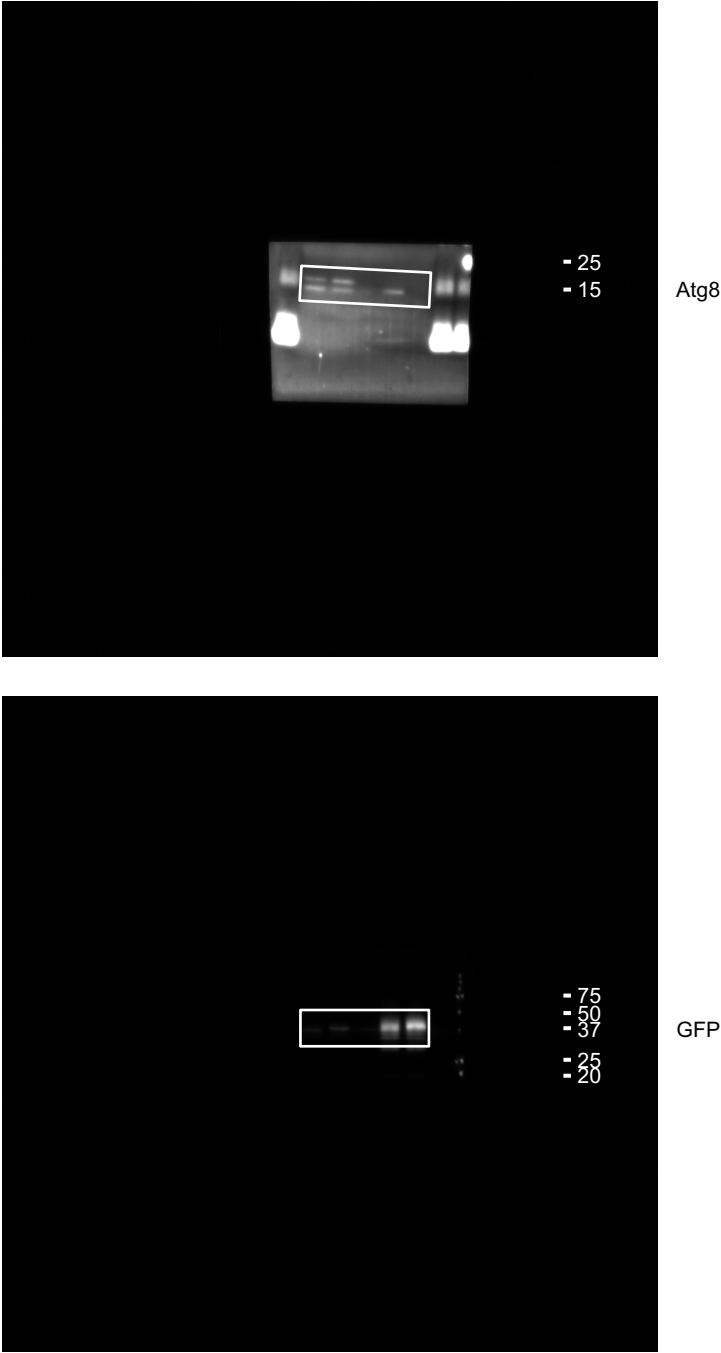

Supplement: Supplementary file 10 — Source data Fig. 4 [file 44318_2024_91_MOESM10_ESM.zip › EMBOJ-2023-115374R1_SourceDataForFig4/4A/4A.pdf]

Figure 5A

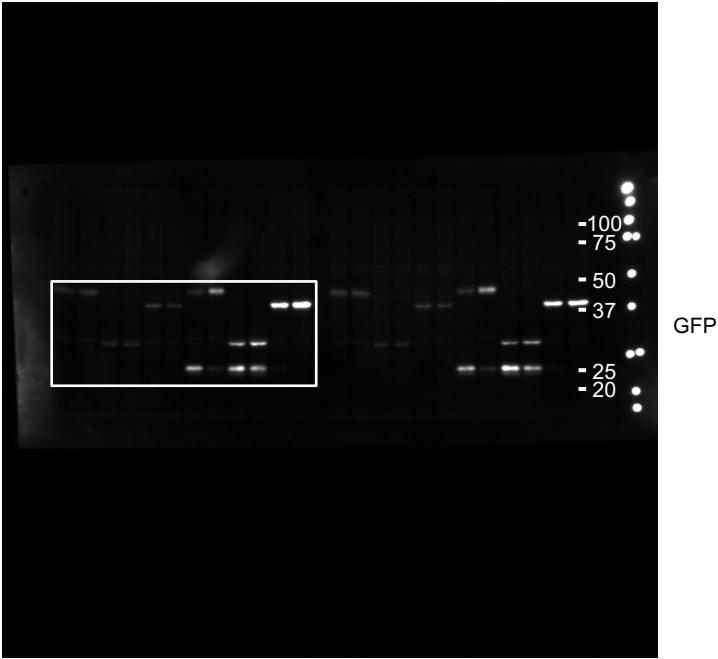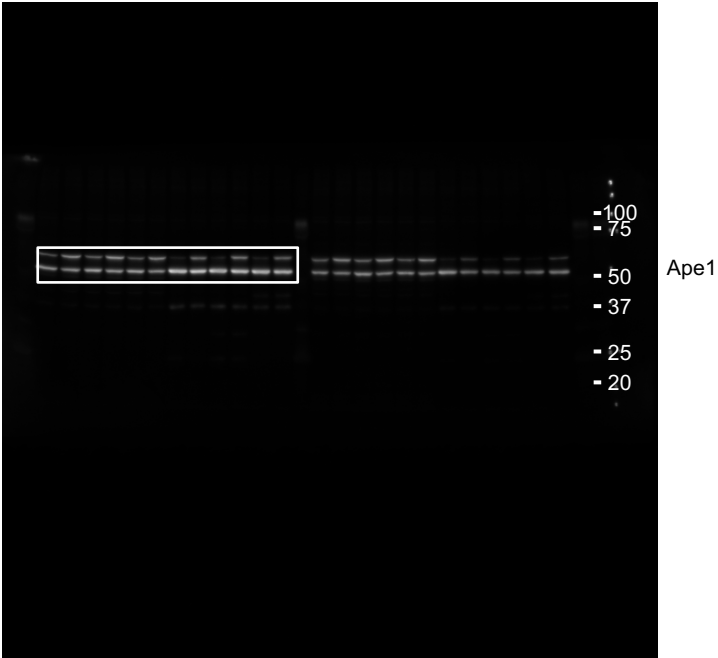

Supplement: Supplementary file 11 — Source data Fig. 5 [file 44318_2024_91_MOESM11_ESM.zip › EMBOJ-2023-115374R1_SourceDataForFig5/5A/5A.pdf]

Figure 5C

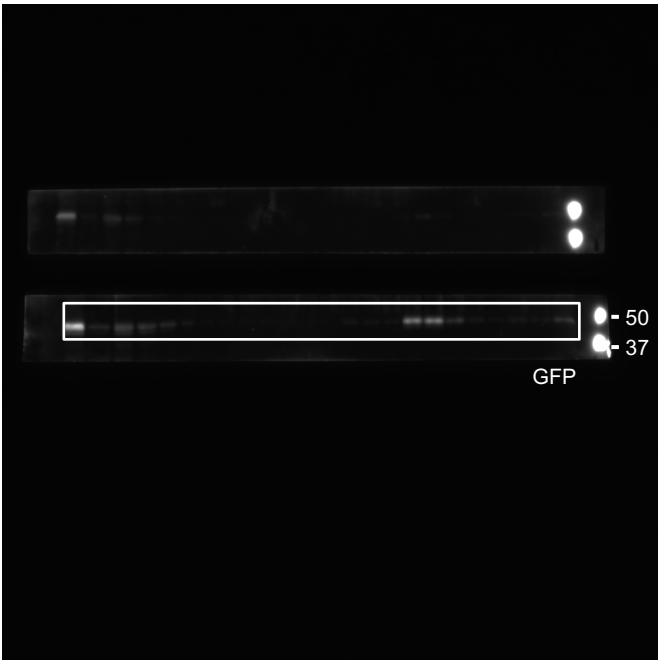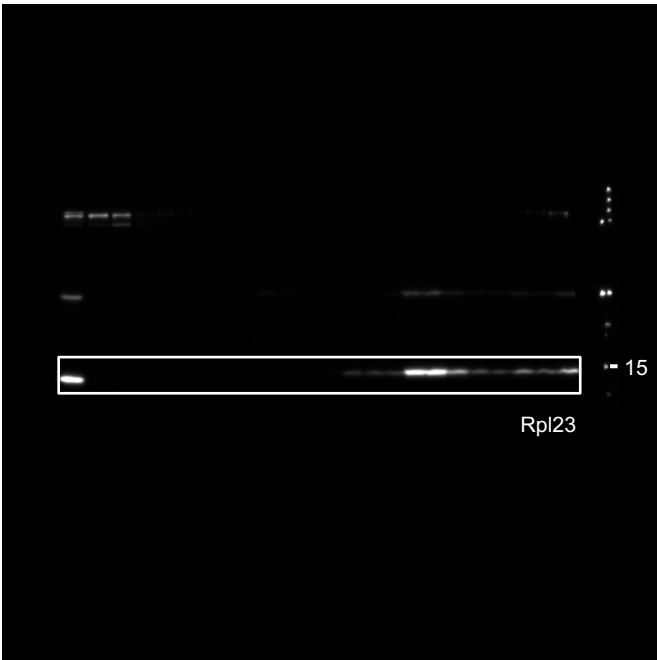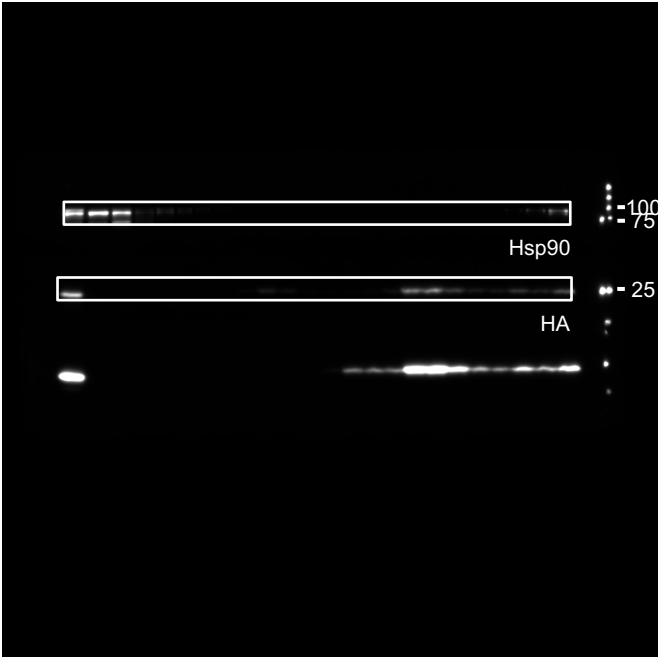

Supplement: Supplementary file 11 — Source data Fig. 5 [file 44318_2024_91_MOESM11_ESM.zip › EMBOJ-2023-115374R1_SourceDataForFig5/5C/5C.pdf]

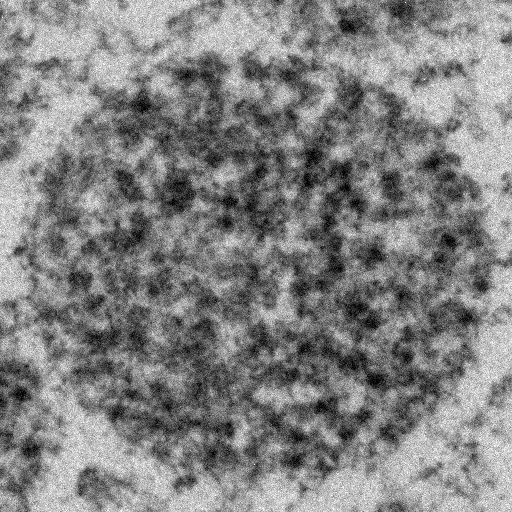

Supplement: Supplementary file 11 — Source data Fig. 5 [file 44318_2024_91_MOESM11_ESM.zip › EMBOJ-2023-115374R1_SourceDataForFig5/5E/2, hab1.tif]

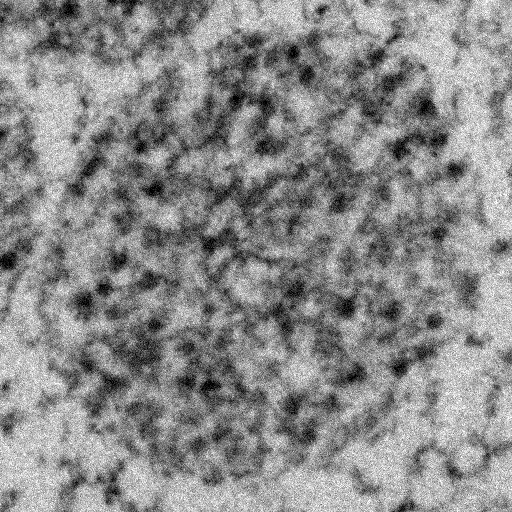

Supplement: Supplementary file 11 — Source data Fig. 5 [file 44318_2024_91_MOESM11_ESM.zip › EMBOJ-2023-115374R1_SourceDataForFig5/5E/1, WT.tif]

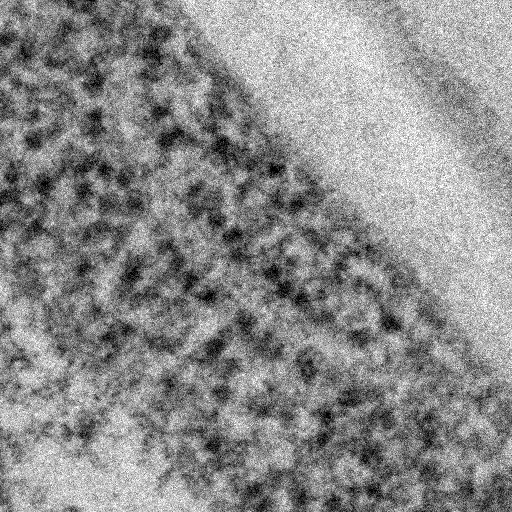

Supplement: Supplementary file 11 — Source data Fig. 5 [file 44318_2024_91_MOESM11_ESM.zip › EMBOJ-2023-115374R1_SourceDataForFig5/5E/RER.tif]

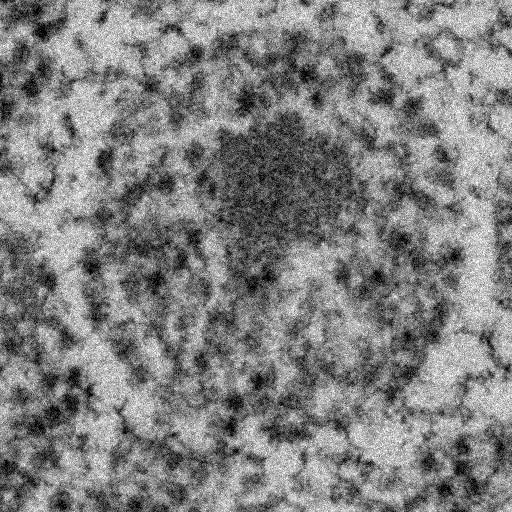

Supplement: Supplementary file 11 — Source data Fig. 5 [file 44318_2024_91_MOESM11_ESM.zip › EMBOJ-2023-115374R1_SourceDataForFig5/5E/3, OE HAB1.tif]

Figure 5B

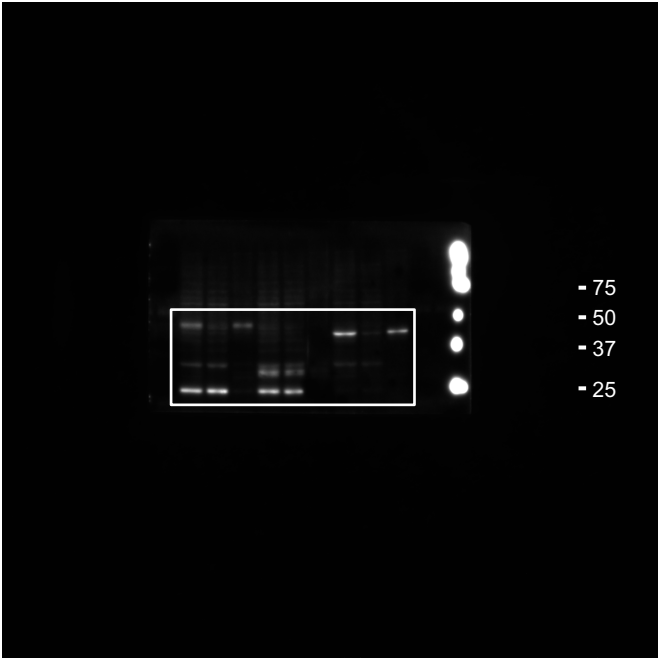

GFP

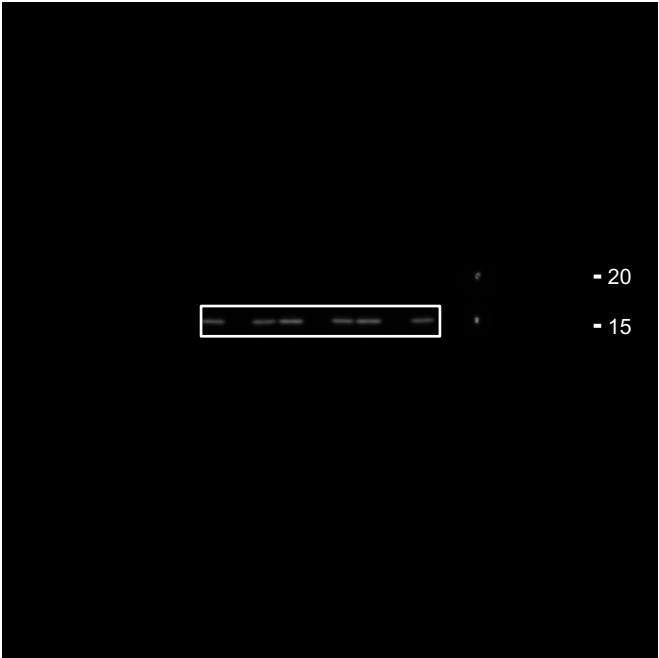

Rpl23

Supplement: Supplementary file 11 — Source data Fig. 5 [file 44318_2024_91_MOESM11_ESM.zip › EMBOJ-2023-115374R1_SourceDataForFig5/5B/5B.pdf]

Figure 6F

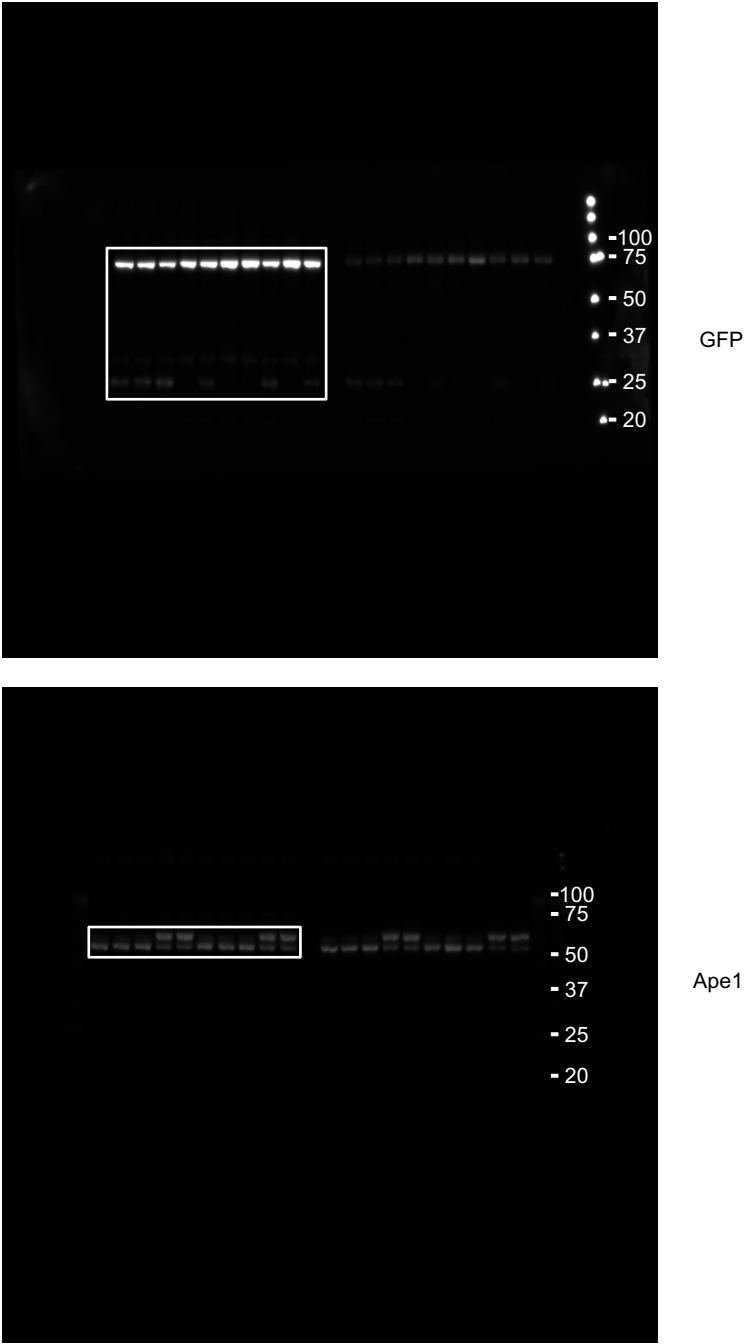

Supplement: Supplementary file 12 — Source data Fig. 6 [file 44318_2024_91_MOESM12_ESM.zip › EMBOJ-2023-115374R1_SourceDataForFig6/6F/6F.pdf]

Figure 6E

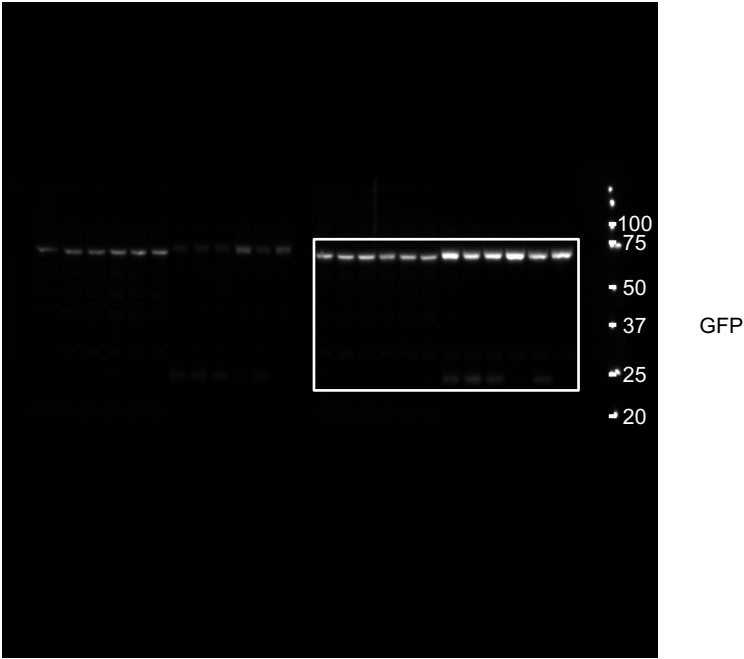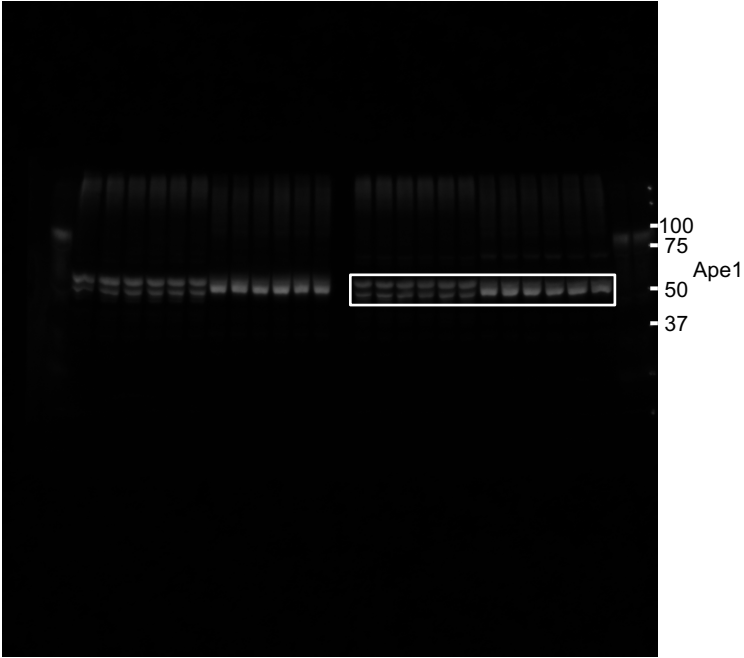

Supplement: Supplementary file 12 — Source data Fig. 6 [file 44318_2024_91_MOESM12_ESM.zip › EMBOJ-2023-115374R1_SourceDataForFig6/6E/6E.pdf]

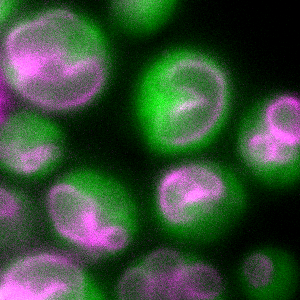

Supplement: Supplementary file 12 — Source data Fig. 6 [file 44318_2024_91_MOESM12_ESM.zip › EMBOJ-2023-115374R1_SourceDataForFig6/6C/1, Hab1.tif]

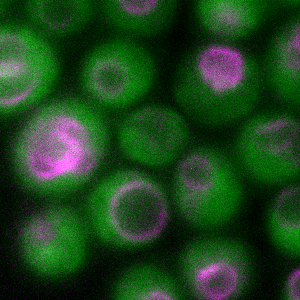

Supplement: Supplementary file 12 — Source data Fig. 6 [file 44318_2024_91_MOESM12_ESM.zip › EMBOJ-2023-115374R1_SourceDataForFig6/6C/4, Hab1(1-42)-GBP atg2.tif]

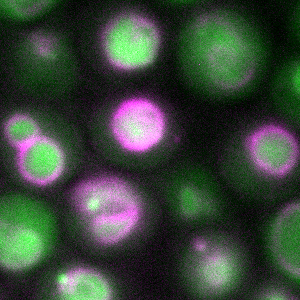

Supplement: Supplementary file 12 — Source data Fig. 6 [file 44318_2024_91_MOESM12_ESM.zip › EMBOJ-2023-115374R1_SourceDataForFig6/6C/2, Hab1(1-42)-GBP.tif]

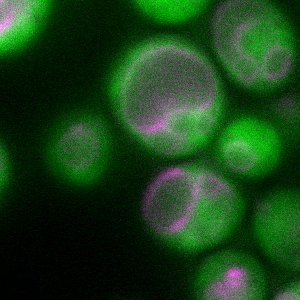

Supplement: Supplementary file 12 — Source data Fig. 6 [file 44318_2024_91_MOESM12_ESM.zip › EMBOJ-2023-115374R1_SourceDataForFig6/6C/3, Hab1(1-42)-GBP3A.tif]
