# Supplementary material for: Receptor-mediated cargo hitchhiking on bulk autophagy
Source: EMBO J. 2024 May 16;43(15):3. doi: 10.1038/s44318-024-00091-8 (PMC11294605; doi:10.1038/s44318-024-00091-8)
Supplement: Supplementary file 14 — Expanded View Figures [file 44318_2024_91_MOESM14_ESM.pdf]

Expanded View Figures

Figure EV1. Fluorescence microscopy of Hab1-GFP (related Fig. 2B).

(**A**) Fluorescence micrographs of WT or *atg1Δ* cells expressing Hab1-GFP and Vph1-2xmCherry. Scale bar, 5 µm. (**B**) Fluorescence micrographs of Hab1-GFP and mCherry-Atg8-expressing cells after treatment with rapamycin for 1 or 4 h. Arrowheads show colocalization. Scale bar, 5 µm. (**C**) Time-lapse imaging of cells expressing Hab1-GFP and mCherry-Atg8. The time after rapamycin addition at which each snapshot was obtained is noted in the upper left corner of each. Arrowheads indicate mCherry-Atg8 or Hab1-GFP puncta. Scale bar, 5 µm.

© The Author(s)

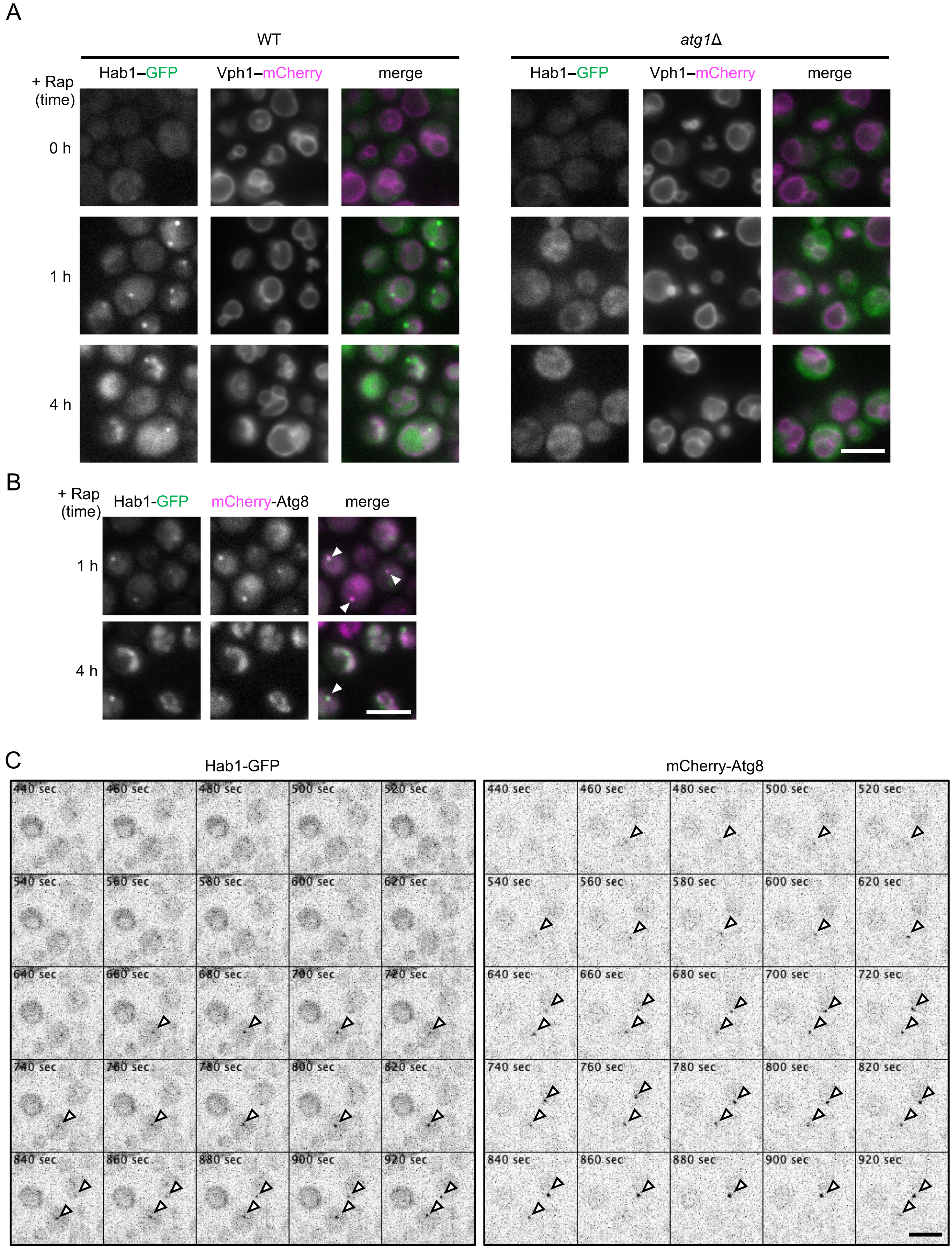
A
WT
atg1Δ
Hab1–GFP
Vph1–mCherry
merge
+ Rap
(time)
0 h
1 h
4 h
B
+ Rap
(time)
Hab1-GFP
mCherry-Atg8
merge
1 h
4 h
C
Hab1-GFP
mCherry-Atg8
440 sec 460 sec 480 sec 500 sec 520 sec
540 sec 560 sec 580 sec 600 sec 620 sec
640 sec 660 sec 680 sec 700 sec 720 sec
740 sec 760 sec 780 sec 800 sec 820 sec
840 sec 860 sec 880 sec 900 sec 920 sec

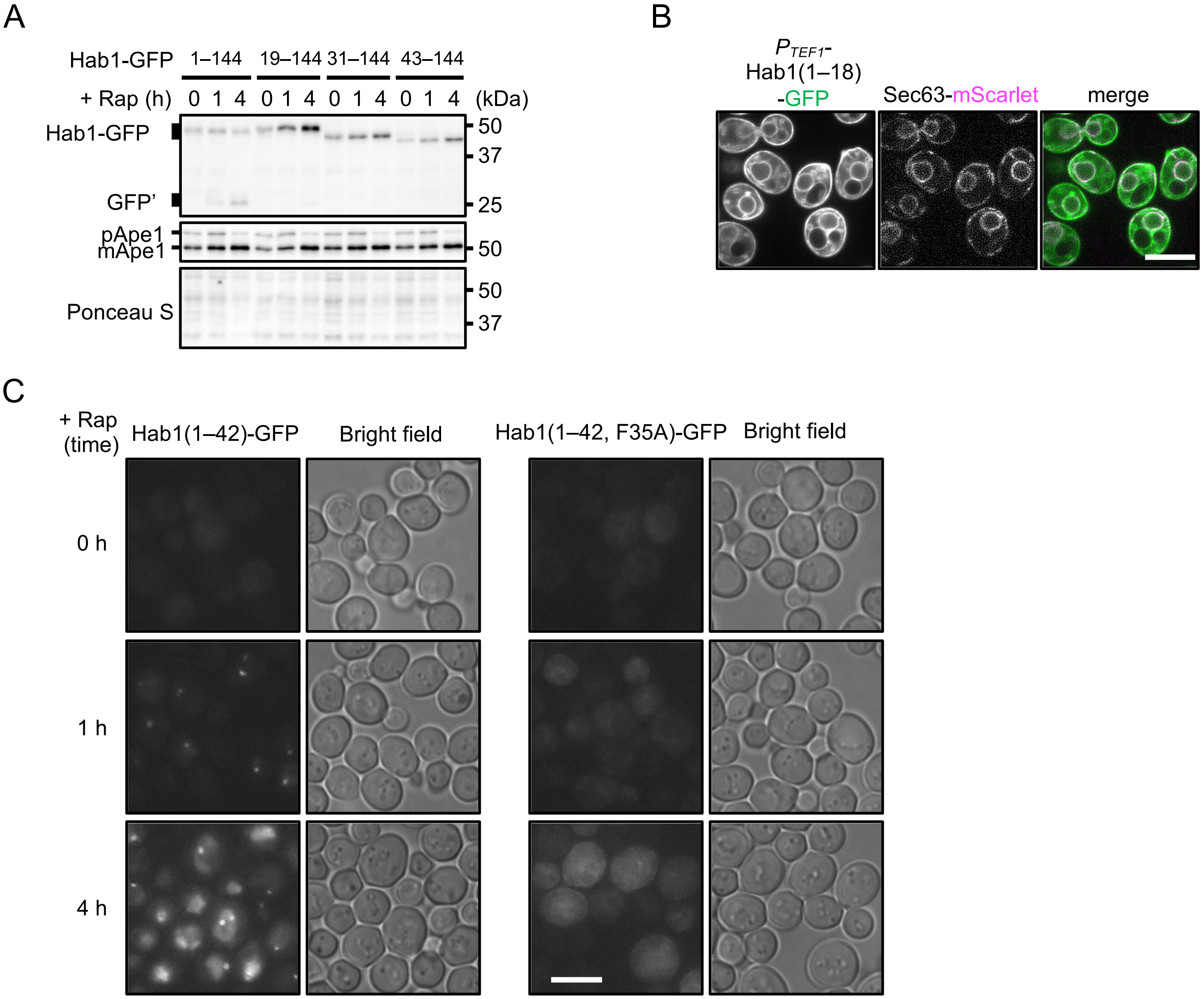

Figure EV2. Hab1 N-terminal helix mutants (related to Fig. 3).

(**A**) GFP cleavage of WT and N-terminal truncation mutants of Hab1. (**B**) Fluorescence micrographs of Hab1-GFP and Sec63-mScarlet (ER-membrane marker) observed by spinning disc confocal fluorescence microscopy. Scale bar, 5 μm. (**C**) Fluorescence micrographs of Hab1(1–42)-GFP-expressing cells. Scale bar, 5 μm.

 © The Author(s)

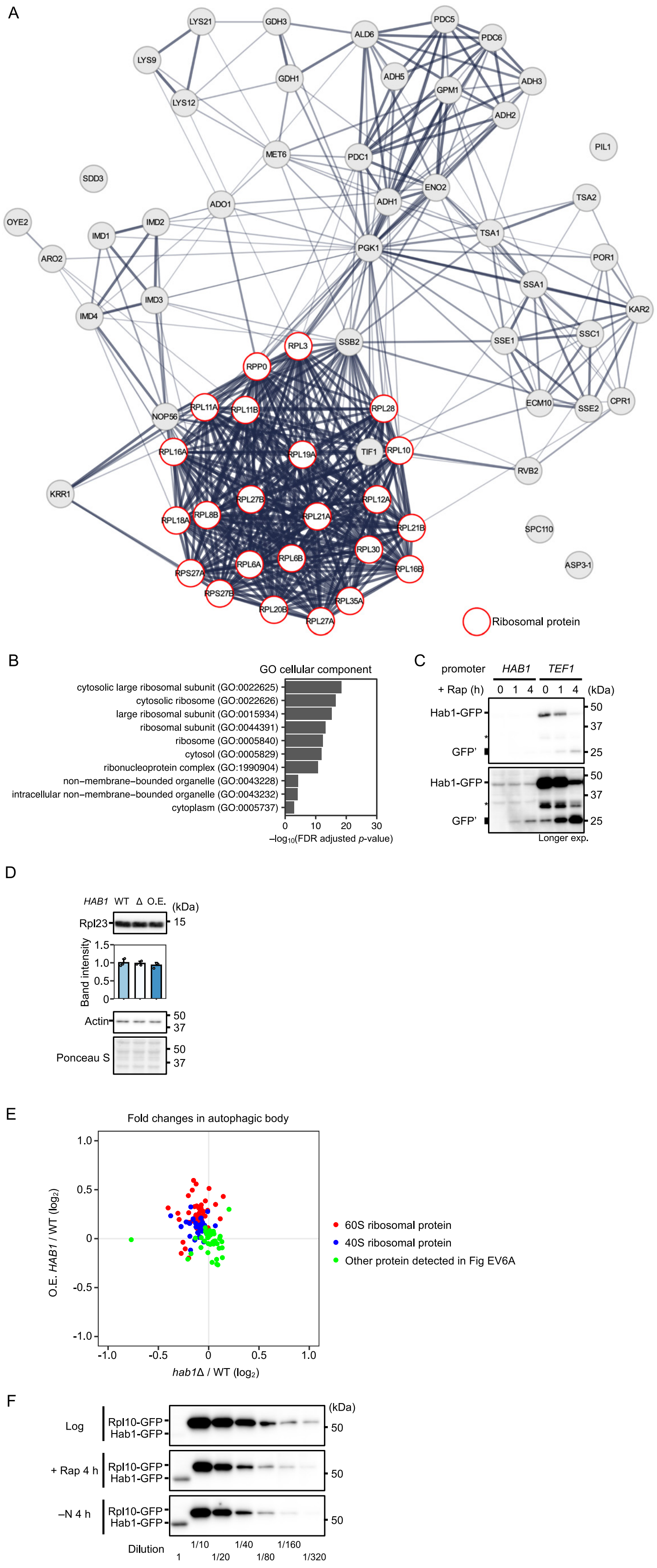

A
Ribosomal protein
B
GO cellular component
cytosolic large ribosomal subunit (GO:0022625)
cytosolic ribosome (GO:0022626)
large ribosomal subunit (GO:0015934)
ribosomal subunit (GO:0044391)
ribosome (GO:0005840)
cytosol (GO:0005829)
ribonucleoprotein complex (GO:1990904)
non-membrane-bounded organelle (GO:0043228)
intracellular non-membrane-bounded organelle (GO:0043232)
cytoplasm (GO:0005737)
−log10(FDR adjusted p-value)
C
promoter HAB1 TEF1
+ Rap (h) 0 1 4 0 1 4 (kDa)
Hab1-GFP
GFP'
Longer exp.
D
HAB1 WT Δ O.E. (kDa)
Rpl23
Band intensity
Actin
Ponceau S
E
Fold changes in autophagic body
O.E. HAB1 / WT (log2)
hab1Δ / WT (log2)
60S ribosomal protein
40S ribosomal protein
Other protein detected in Fig EV6A
F
Log
+ Rap 4 h
−N 4 h
Rpl10-GFP
Hab1-GFP
(kDa)
Dilution
1 1/10 1/20 1/40 1/80 1/160 1/320

Figure EV3. Hab1 binds to ribosome (related to Fig. 5).

(**A**) A network map of Hab1(43–144)-interacting proteins. Proteins immunoprecipitated with Hab1(43–144)-GFP were detected by LC-MS/MS. Detected Hab1(43–144)-GFP interacting proteins were analyzed using STRING (Szklarczyk et al, 2019) after control sample (GFP-immunoprecipitated) proteins were excluded. (**B**) GO enrichment analysis of Hab1-interacting proteins. Analysis was performed on the cellular component for all proteins identified in (A) using the GO Consortium (accessed August, 2022) (Ashburner et al, 2000). GOs with FDR-adjusted *p*-values lower than 0.01 are indicated. FDR was calculated using the one-way Fisher's exact test. (**C**) Confirmation of overexpression of Hab1 by placing *HAB1* under the control of the *TEF1* promoter. Cells expressing Hab1-GFP under the control of the *HAB1* or *TEF1* promoter were subjected to immunoblotting. Cells were cultured in SD media to the logarithmic growth phase and then treated with rapamycin to induce autophagy. (**D**) The effect of Hab1 on ribosome biosynthesis was evaluated by quantification of Rpl23. There was no detectable change in ribosomal protein levels in *atg15*Δ, *atg15*Δ *hab1*Δ, and *HAB1*-overexpressing *atg15*Δ cells in the logarithmic growth phase on YPD. Quantifications of band intensities are shown as mean ± SD. ($n = 3$); data are normalized to the WT band. (**E**) An analysis of non-ribosomal proteins in autophagic bodies detected in Fig. EV3A. Fold changes are shown as normalized label-free quantification of each 40 S or 60S ribosomal proteins in autophagic bodies isolated from *hab1*Δ (x-axis) or *HAB1*-overexpressing cells (y-axis) relative to that of WT cells. Green: non-ribosomal proteins detected in Fig. EV3A, red: 60S ribosomal proteins, blue: 40S ribosomal proteins. The same data set as shown in Fig. 5D was used. (**F**) Examination of abundance ratios of Hab1 to ribosomes by immunoblotting. Hab1-GFP- or Rpl10-GFP-expressing *atg2*Δ cells were cultured in YPD media to the logarithmic phase of growth and then treated with rapamycin or cultured in SD–N medium for 4 h. Cells were then harvested. For Rpl10-GFP-expressing cells, diluted samples were applied at the ratios described below each lane.

 © The Author(s)

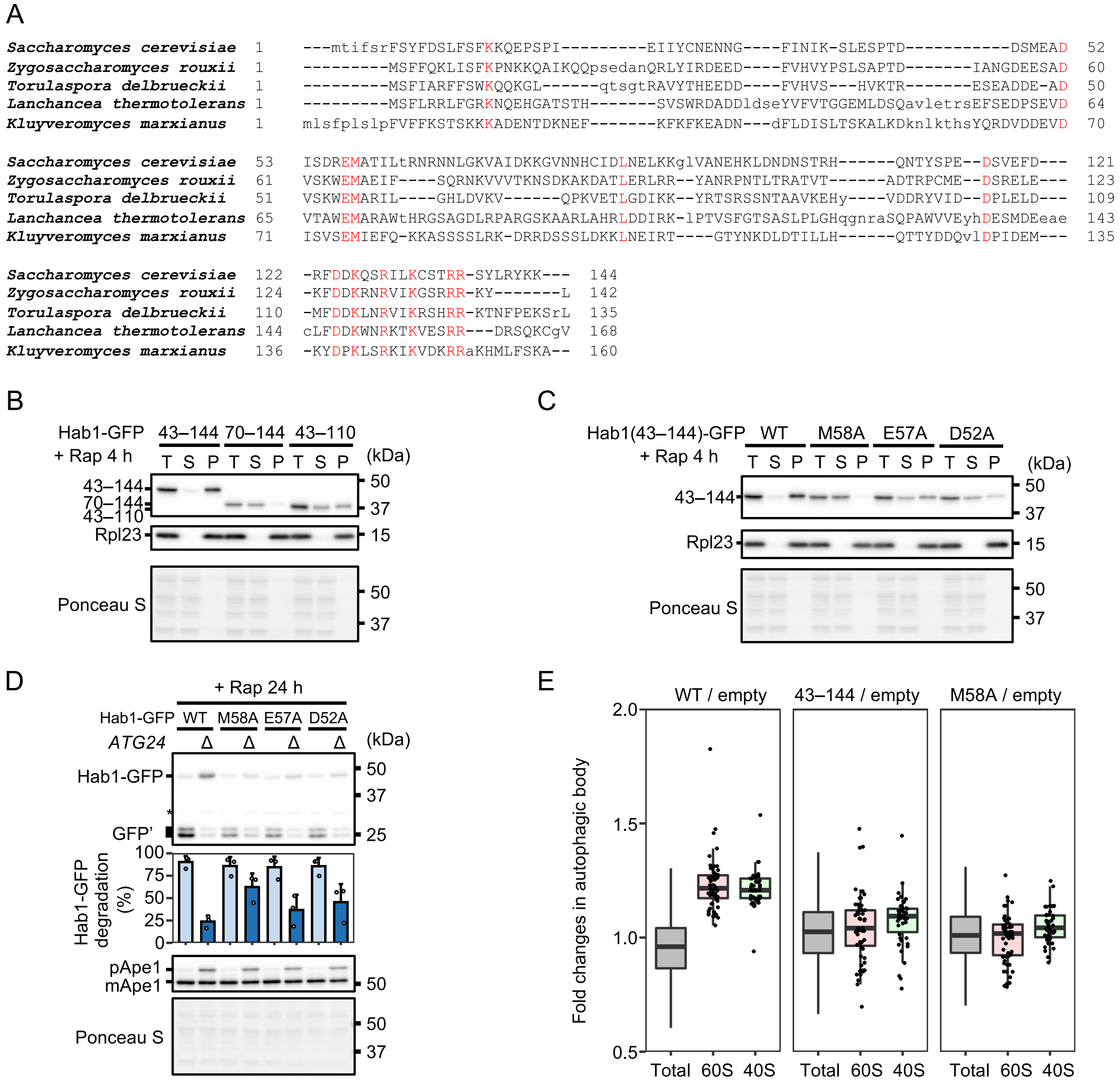

Figure EV4. The N- and C-terminal regions of Hab1 are important for the Hab1-dependent delivery of ribosomes to vacuoles.

(**A**) Hab1 orthologs of *Zygosaccharomyces rouxii*, *Torulaspora delbrueckii*, *Lanchancea thermotolerans,* and *Kluyveromyces marxianus* were identified based on sequence homology using PSI-BLAST searches. Amino acid sequences are shown. The alignment was performed using COBALT (https://www.ncbi.nlm.nih.gov/tools/cobalt/). Conserved residues are shown in red. (**B**) Immunoblotting of subcellular fractions. Lysates were obtained from cells expressing Hab1-GFP truncates. (**C**) Immunoblotting of subcellular fractions. Lysates were obtained from cells expressing Hab1-GFP point mutants. (**D**) GFP cleavage of Hab1 point mutants in WT and *atg24*Δ cells. The percentages of GFP′ to total GFP (GFP′ + full-length GFP) are shown as mean ± SD ($n = 3$). (**E**) Proteomic analyses of ribosomal proteins in autophagic bodies. Autophagic bodies isolated from *hab1*Δ *atg15*Δ cells harboring empty vector, *HAB1*(WT), *HAB1*(43-144) or *HAB1*(M58A) following 6 h rapamycin treatment were subjected to proteomic analyses. Fold changes are shown as normalized label-free quantifications of each 40S or 60S ribosomal protein in autophagic bodies. "Total" indicates all detected proteins. Box plots are shown as median (middle bar) with 25th and 75th percentiles and 1.5 × interquartile in whiskers.

© The Author(s)

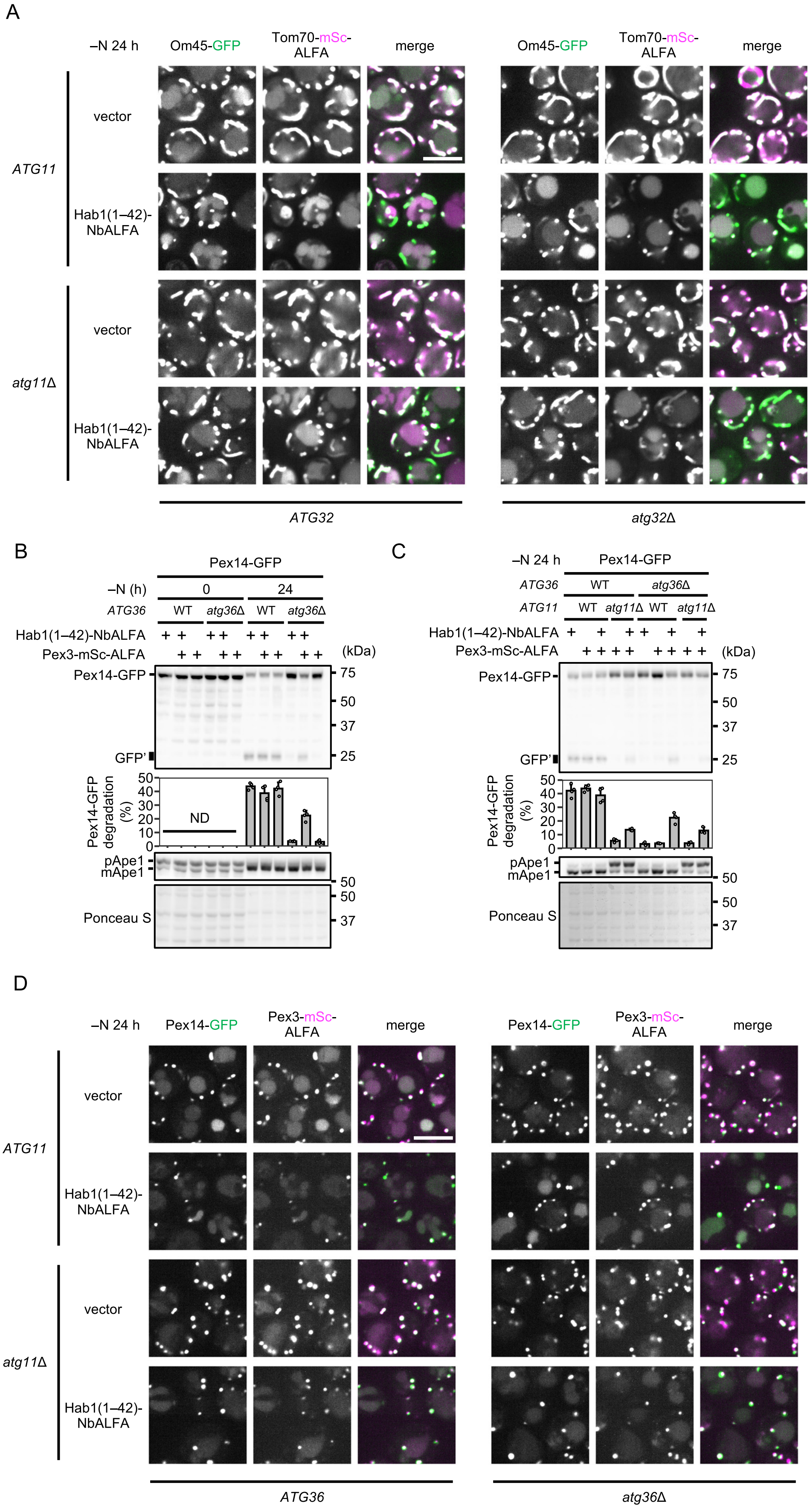
A
–N 24 h
Om45-GFP
Tom70-mSc-ALFA
merge
vector
Hab1(1–42)-NbALFA
ATG11
atg11Δ
ATG32
atg32Δ
B
Pex14-GFP
–N (h)
0
24
ATG36
WT
atg36Δ
Hab1(1–42)-NbALFA
Pex3-mSc-ALFA
(kDa)
75
50
37
25
GFP'
Pex14-GFP degradation (%)
ND
pApe1
mApe1
Ponceau S
C
ATG11
atg11Δ
D
Pex14-GFP
Pex3-mSc-ALFA
ATG36
atg36Δ

© The Author(s)

Figure EV5. Binding to Hab1(1–42) allows receptor- and scaffold protein-independent delivery of organelles to vacuoles (related to Fig. 6).

(**A**) Fluorescence microscope images of Hab1(1–42)-bound mitochondria showing delivery to the vacuole. Om45-GFP-expressing cells were observed 24 h after shifting to SD–N medium. Scale bar, 5 μm. (**B**) Rates of peroxisomal delivery to the vacuole were examined by GFP cleavage of Pex14-GFP. Hab1(1–42) was tethered to mitochondria using the ALFA-tag system. –N, nitrogen starvation; Percentages of GFP′ to total GFP (GFP′ + full-length GFP) are showns as mean ± SD ($n = 3$). ND, not determined. Blot data are representative of two independent experiments. (**C**) Rates of peroxisomal delivery to the vacuole were examined by GFP cleavage of Pex14-GFP. Percentages of GFP′ to total GFP (GFP′ + full-length GFP) are shown as mean ± SD ($n = 3$). A subset of data is derived from samples used for quantification in (**B**). Blot data are representative of three independent experiments. (**D**) Fluorescence micrographs during nitrogen starvation (related to Figs. EV5B and 5C). Scale bar, 5 μm.
